# The effect of long-lasting insecticidal nets (LLINs) physical integrity on utilization

**DOI:** 10.1186/s12936-021-03976-9

**Published:** 2021-12-18

**Authors:** Honelgn Nahusenay Hiruy, Ayele Zewde, Seth R. Irish, Semira Abdelmenan, Adugna Woyessa, Yonas Wuletaw, Hiwot Solomon, Mebrahtom Haile, Achamyelesh Sisay, Sheleme Chibsa, Alemayehu Worku, Josh Yukich, Yemane Berhane, Joseph Keating

**Affiliations:** 1 Tulane University School of Public Health and Tropical Medicine, New Orleans, LA USA; 2 Addis Continental Institute of Public Health, Addis Ababa, Ethiopia; 3 Center for Disease Control (CDC), Atlanta, USA; 4 US President’s Malaria Initiative (PMI), Addis Ababa, Ethiopia; 5 Ethiopian Public Health Institute, Addis Ababa, Ethiopia; 6 Ethiopia Federal Ministry of Health, Addis Ababa, Ethiopia

## Abstract

**Background:**

In Ethiopia, despite improvements in coverage and access, utilization of long-lasting insecticidal nets (LLINs) remains a challenge. Different household-level factors have been identified as associated with LLIN use. However, the contribution of LLIN physical integrity to their utilization is not well investigated and documented. This study aimed to assess the association between the physical integrity of LLINs and their use.

**Methods:**

This study employed a nested case-control design using secondary data from the Ethiopian LLIN durability monitoring study conducted from May 2015 to June 2018. LLINs not used the night before the survey were identified as cases, while those used the previous night were categorized as controls. The physical integrity of LLINs was classified as no holes, good, acceptable, and torn using the proportionate hole index (pHI). A Generalized Estimating Equation (GEE) model was used to assess and quantify the association between LLIN physical integrity and use. The model specifications included binomial probabilistic distribution, logit link, exchangeable correlation matrix structure, and robust standard errors. The factors included in the model were selected first by fitting binary regression, and then by including all factors that showed statistical significance at *P*-value less than 0.25 and conceptually relevant variables into the multivariate regression model.

**Results:**

A total of 5277 observations fulfilled the inclusion criteria. Out of these 1767 observations were cases while the remaining 3510 were controls. LLINs that were in torn physical condition had higher odds (AOR [95% CI] = 1.76 [1.41, 2.19]) of not being used compared to LLINs with no holes. Other factors that showed significant association included the age of the LLIN, sleeping place type, washing status of LLINs, perceptions towards net care and repair, LLIN to people ratio, economic status, and study site.

**Conclusion and recommendation:**

LLINs that have some level of physical damage have a relatively higher likelihood of not being used. Community members need to be educated about proper care and prevention of LLIN damage to delay the development of holes as long as possible and use available LLINs regularly.

## Background

In Ethiopia, despite improvements in coverage and access, utilization of long-lasting insecticidal nets (LLINs) remains a challenge [1, 2]. Coverage (the proportion of households with at least one LLIN) increased from 55% to 2011 [3], to 64% in 2015 [1], and 67% in 2020 [4]. However, not all LLINs owned by a household were used. For example, according to the 2020 national LLIN ownership, utilization, and malaria treatment-seeking behaviour survey, the proportion of LLINs that were used the night before the survey was 48% [4]. LLINs use might be affected by several factors, including external factors (i.e., malaria transmission settings, residence being urban or rural), behavioural factors (i.e., knowledge and attitudes), housing conditions (i.e., numbers and types of sleeping spaces, number of people in the house), and net level factors (i.e., physical integrity, shape, colour, cleanness, age) which are discussed below.

External factors have been identified to be important determinants for the utilization of available LLINs. For example, a study done in one district in Ethiopia showed that being outside the malaria transmission season was the main reason cited by 69.7% of the households for not using their LLINs the previous night [5]. Other studies have identified other external factors, such as distance from mosquito breeding sites [6], proximity to a health facility [7], altitude, availability of other interventions, such as indoor residual spraying (IRS) [8], and being an urban or rural residence [9] to be associated with the use of available LLINs.

Behavioural factors are also important predictors for the utilization of available LLINs. In Ethiopia knowledge of malaria and LLINs [10, 11], erroneous perception about LLINs (including evaluating insecticidal efficacy of LLINs based on their ability to kill bed bugs, and tending not to use LLINs that do not kill bed bugs and other arthropods) [12], and exposure to malaria-related messages [13] were associated with utilization of available LLINs. Risk perception about malaria was also associated with use of LLINs in other places [14]. In 2015, a study by Birhanu et al. in southwestern Ethiopia revealed that about a third (30%) of respondents did not sleep under a LLIN the night preceding the survey due to behaviour-driven factors such as low risk perception and perception of low efficacy of LLINs [9].

Household characteristics have also been found to be determinants of LLIN use. For example, utilization tends to be higher in households headed by individuals with higher formal education [15, 16]. The effect of a household’s economic status on LLIN utilization seems mixed. Some studies reported populations in the highest wealth quintile are highly likely to use their LLINs, while others report the opposite [17, 18]. Some studies reported no significant association between household wealth and LLIN use [19]. Other household characteristics, such as family size [16] and the number of sleeping places [10] were also found to be associated with LLIN use.

Characteristics of LLINs such as size, shape, colour, age, and level of damage have also been associated with use. LLINs that were big enough to fit the sleeping space were found to be associated with higher rates of use in Uganda [20]. LLINs that were more than 1 year old were less likely to be used in several areas of Ethiopia [8]. Conical-shaped LLINs were preferred and more likely to be used than rectangular ones in Ethiopia [12, 13, 21]. Bed nets that matched the preferred colour of individual users were more likely to be used in Malawi [22].

A limited number of studies have assessed the relationship between LLIN physical integrity and LLIN use in Ethiopia. In a qualitative study done in Northwest Ethiopia, “early damage” of LLINs was reported to be associated with non-persistent use. However, the level of damage and association was not quantified [12]. In 2011, Ngondi et al. analysed a cross-sectional survey done in 2006 and the 2007 Ethiopia malaria indicator survey. Their findings indicated that LLINs in fair condition (no holes > 33 mm diameter) were more likely to be used compared to nets in bad/torn conditions (having 5 or more holes > 33 mm diameter) [8]. In 2012, Batiso et al. reported 46% of bed nets were not used when they were considered too old or torn. However, their measurement of physical condition revealed that the bed nets considered to be too torn to use had similar levels of damage with others assumed to be in good condition [23].

These studies leave several gaps that need to be addressed. They were limited to small geographical areas or in one malaria transmission setting. However, as Ethiopia is a country with heterogeneous malaria transmission, assessing the effect of LLIN physical integrity on utilization in different settings might reveal important differences. Two studies [8, 23] tried to assess the association between the physical integrity of LLINs and their use, however, both studies were done before the standardization of LLIN physical integrity measurement. Qualitative studies have identified important concepts such as a household’s inclination not to use damaged LLINs but there is a need to quantify this association. To fill these gaps, this study aimed to assess the association between physical integrity and utilization of LLINs in different malaria transmission settings by analysing data collected as part of the Ethiopian LLIN durability monitoring study done from May 2015 to June 2018, that measured the physical integrity of LLINs using a standardized method.

## Methods

### Study setting

This study was done in four study sites namely Amhara, Oromia, Tigray, and Southern Nations, Nationalities, and People’s Region (SNNPR), of Ethiopia that constitute about 86% of the total population of the country. They represent the country’s different malaria transmission settings, which are heterogeneous, seasonal, and characterized by the co-existence of *Plasmodium falciparum* and *Plasmodium vivax*. *Plasmodium falciparum* accounted for ~ 60% of cases (range 55–69%) and *P. vivax* 40% (range 31–45%) from 2001 to 2016 [2]. *Anopheles arabiensis* is the primary vector with *Anopheles pharoensis*, *Anopheles coustani*, *Anopheles funestus, Anopheles nili* (and recently *Anopheles stephensi*) having a minor role in malaria transmission [24]. The main malaria prevention and control interventions are LLINs, indoor residual spraying (IRS), larvae source management (LSM), prompt diagnosis and early treatment of cases, and behavioural change communication [25]. In 2015, the country distributed close to six million LLINs, out of which the two brands of LLIN, namely MAGNet® (VKA Polymers, Tamil Nadu, India) and PermaNet® 2.0 (Lausanne, Switzerland) take 69.0% and 17.9% of the share. Both brands were distributed in each region [26].

### Study design

A nested case-control design was used to assess the association between the physical integrity of LLINs and their utilization. Case and control designations were defined based on the reported utilization of LLINs the night before the survey. LLINs used the night before the survey were considered as controls while those not used were categorized as cases.

### Sample size and power

The parent study measured the utilization of LLINs at four-time points. A total of 5277 observations fulfilled the inclusion criteria. Out of these 1767 observations were cases (LLINs not used) while the remaining 3510 were controls (LLINs used). The proportion of exposure (i.e., torn LLINs) among controls was 9.8%. With this level of exposure, assumption of 95% confidence level, and 80% power, the sample size was adequate to detect a minimum odds ratio of 1.167.

### Sampling procedures

At baseline, the study enrolled a total of 1840 households. These households were selected using a multistage sampling procedure. Each of the four study sites (i.e., regions) was treated as a separate sampling domain. For each of the study sites, a list was prepared of all districts that had received nets in the past 2 months or expected to receive nets before data collection. Furthermore, the districts were categorized based on their malaria transmission stratification status. A total of 12 districts were selected: three from high Annual Parasite Incidence (API) >= 100/1000), seven from moderate (API = 5–100/1000), and two from low (API < 5/1000) transmission settings. Selection of clusters (i.e., enumeration areas (EA)) was done proportional to the size of the districts’ population. A total of 92 clusters were selected randomly across all study sites. The selection of households was done using systematic random sampling. First, a complete listing of all the households within the selected EAs was done to generate the sampling frame. Using this sampling frame, twenty households were selected from each cluster using systematic random sampling. All LLINs in the household that had been received during the 2015 campaign were recruited in the study. They were tagged using a plastic coin with engraved numbers and monitored annually for 3 years.

### Data collection procedure

Data were collected using two methods: interviews with heads of households and direct observation of LLINs. The first method was used to gather information about household characteristics such as the socio-economic status (SES) of households; knowledge, attitudes and behaviour regarding malaria and bed nets, as well as other variables, such as LLIN handling practices. The second method was used to assess the physical integrity of LLINs by measuring the presence, size, and location of holes. Electronic data collection using Open Data Kit (ODK) was used to collect and transfer data from the field to a central database [27]. Data collection was done before the beginning of the wet season (May and June).

### Measurements

The exposure variable, the physical integrity of LLINs, was measured using World Health Organization (WHO) guidelines that outline how to monitor the durability of LLINs in field settings [28]. Since all LLINs were brand new at the time of distribution, significant damage was not anticipated within the first 2 months post-distribution, hence physical inspection was not done during baseline data collection. The level of damage was measured by physical inspection during the follow-up surveys 12, 24, and 36 months after LLIN distribution. The LLINs were taken outdoors and placed over a metal frame, where they could be examined more accurately for holes and other types of damage. The holes (including tears in the netting and split seams) were measured using a measuring tape and their sizes and locations were recorded. The diameter of holes was measured in the longest dimensions. Location was identified by dividing the LLIN as zones one (lower one-third), two (the middle one-third), and three (top one-third) and roof. In addition, evidence of repairs and types of repairs were recorded.

A composite indicator called the proportionate hole index (pHI) was used to quantify the level of damage [28]. The pHI was calculated by weighting each hole by size and summing for each net. First, each hole was categorized as either hole size one (0.5 – < 2.0 cm), two (2 – < 10 cm), three (10 – 25 cm) or four (> 25 cm). Holes with a diameter below 0.5 cm were ignored, as these were considered too small for a mosquito to easily pass through. Then, each hole was weighted according to the average area of each hole category, with weights of 1, 23, 196, and 578 applied for hole sizes one, two, three, and four, respectively. Then, the physical condition of LLIN was placed into one of four categories: no holes, good (total hole surface area < 0.01 m² or pHI < 64), serviceable (total hole surface area ≤ 0.1 m² or pHI 64 – 642 ), and torn (total hole surface area > 0.1 m² or pHI > 642) [28].

The outcome variable, utilization of LLINs, was measured by asking the owners if it was used the previous night. LLINs that were used were classified as controls and coded as zero (0) while those that were not used were categorized as cases and coded as one (1). This variable was measured four times for a given LLIN, at each round of data collection. The LLINs to people ratio was calculated by dividing the total number of LLINs (both cohort and non-cohort) by the total number of household members who stayed in the house the night before the survey.

Perceptions towards net care and repair were measured by presenting a series of eight Likert-scale statements to the respondent. Each response was categorized into one of three categories: negative, positive, and very positive. Details of the method were described in a previous publication using the baseline data [29].

The economic status of households was measured based on a composite measure of wealth index based on household assets and housing conditions [30].

### Data analysis

A subset of data was prepared from the parent study by excluding observations that had missing or unknown outcomes (i.e., utilization status) or exposures (i.e., the physical integrity of LLINs). Then, the data were analysed using Stata version 15 [31]. A Generalized Estimating Equation (GEE) logistic regression model was used to account for the repeated measures of exposure and outcome. The model specifications included binomial probabilistic distribution, logit link, exchangeable correlation matrix structure, and robust standard errors. The factors to be included in the model were selected first by fitting binary regression, then by including all factors that showed *P-*value less than 0.25 and conceptually relevant variables into the multivariate regression model.

### Ethical considerations

The study protocol was submitted and approved by the Institutional Review Board (IRB) at Addis Continental Institute of Public Health (ACIPH), which is a nationally registered board. Upon approval, permission letters were obtained from the four regions. At the household level, the study was fully explained to respondents and their verbal consent was obtained. Personal identifiers in the survey questionnaire were only used for follow-up purposes and locating the LLINs over the three years.

## Results

### Characteristics of case and control LLINs

A total of 5277 observations fulfilled the inclusion criteria. Of these 1767 observations were cases (LLINs not used) while the remaining 3510 were controls (LLIN used), making the case to control ratio close to 1:2. In both cases and controls, the majority (72.2%) of the LLINs were MAGNet® while the remaining were PermaNet 2.0®. Bed frames made of sticks were the most common type of beds over which of the cases (29.6%) and controls (44.5%) LLINs were used. The proportion of LLINs ever washed was almost equal between cases and controls, 48.8% and 48.1% respectively (see Table 1).

**Table 1:** Characteristics of case and control LLINs

Variables	Control LLINs (used last night) Freq. (%) n = 3510	Case LLINs (not used last night) Freq. (%) n = 1767
LLIN physical integrity		
No holes	2488 (70.9)	1088 (60.7)
Good	321 (9.8)	153 (8.6)
Acceptable	338 (9.4)	187 (11.0)
Torn	363 (9.8)	339 (19.6)
Brand		
MAGNet®	2556 (72.2)	1281 (72.2)
PermaNet 2.0®	954 (27.8)	486 (27.8)
LLIN age		
0 year	1283 (35.5)	209 (13.3)
1 year	1353 (39.1)	662 (36.1)
2 years	659 (19.4)	627 (36.1)
3 years	215 (6.1)	269 (14.5)
Main type of sleeping space net was mostly used		
Bed frame (finished)	664 (19.3)	338 (18.7)
Bed frame (sticks)	1567 (44.5)	522 (29.6)
Foam mattress	143 (4.1)	98 (5.1)
Reed mattress	138 (3.9)	95 (5.7)
Grass mattress	639 (18.0)	141 (8.6)
Floor with no mattress	336 (9.5)	493 (27.6)
Others	23 (0.7)	80 (4.7)
LLIN ever washed	1,668 (48.1)	873 (48.8)
Mean age household head (SD)	47.4 (14.3)	45.17 (13.9)
Household head gender		
Male	2943 (85.1)	1406 (81.8)
Female	514 (14.9)	334 (18.2)
Household head educational status		
No formal education	1790 (51.6)	908 (51.3)
Primary (1–6)	1030 (29.5)	475 (27.4)
Secondary (7–8)	292 (7.9)	150 (8.9)
High school (9–10)	214 (5.9)	120 (6.9)
Above high school	176 (4.9)	94 (5.4)
Ever exposed to net care and repair messages	1110 (30.1)	554 (30.5)
Perception towards net care and repair		
Negative	609 (18.0)	346 (21.5)
Positive	1902 (55.1)	938 (53.7)
Very positive	989 (26.9)	478 (24.8)
Mean family size (SD)	5.69 (2.12)	5.36 (2.03)
Number of rooms used for sleeping		
1	1678 (47.5)	767 (41.2)
2	1470 (42.0)	755 (44.1)
3	307 (8.6)	223 (13.2)
4	48 (1.6)	16 (1.0)
5	7 (0.2)	6 (0.4)
Mean LLIN to people ratio (SD)	0.41 (0.23)	0.49 (0.31)
LLIN to people ratio		
Less than one LLIN for every two people	2352 (67.0)	980 (57.3)
One LLIN for every two people	631 (18.4)	314 (18.1)
More than one LLIN for every two people	522 (14.7)	478 (24.6)
Wealth quintile		
Lowest	839 (23.8)	321 (17.4)
Second	731 (20.7)	355 (20.1)
Middle	717 (20.1)	330 (19.0)
Fourth	679 (19.4)	286 (17.4)
Highest	544 (16.0)	475 (26.0)
Study site		
Tigray	609 (17.7)	845 (45.2)
Amhara	584 (17.6)	280 (16.2)
Oromia	1011 (31.7)	461 (29.6)
SNNPR	1306 (33.0)	181 (8.9)
Malaria transmission setting		
Low (API < 5/1000)	561 (14.3)	522 (24.3)
Moderate (API 5–100/1000)	2773 (81.5)	1138 (70.6)
High (API >= 100/1000	176 (4.3)	107 (5.1)

The majority of LLINs in cases (81.8%) and in controls (85.1%) were owned by households headed by males. A bit more than half of the case (51.3%) and control (51.6%) household heads had no formal education. The proportion of LLINs owned by households whose heads had very positive attitudes towards bed net care and repair was slightly lower among cases (24.8%) compared to controls (26.9%) (Table 1).

### Association between physical integrity of LLINs and use

The final GEE model revealed that the physical integrity of LLINs had a statistically significant association with usage. The odds of LLIN utilization decreased as the physical integrity deteriorated. LLINs that were in torn physical condition had higher odds (AOR [95% CI] = 1.76 [1.41, 2.19]) of not being used compared to those LLINs with no holes (Table 2).

**Table 2:** GEE model assessing association factors with LLINs non-use in Ethiopia

Variables	COR (95% CI)	AOR^+^ (95% CI)
LLIN Physical integrity based on pHI		
No holes	Ref.	Ref.
Good	1.10 (0.88, 1.37)	0.97 (0.76, 1.25)
Acceptable	1.5 1(1.23, 1.84)*	1.23 (0.96, 1.57)
Torn	2.5 5(2.14, 3.04)*	1.76 (1.41, 2.19)*
Brand		
MAGNet®	Ref.	
PermaNet 2.0®	1.00 (0.9, 1.2)	
LLIN age		
0 year	Ref.	Ref.
1 year	2.38 (2.01 2.82)*	3.53 (2.85, 4.37)*
2 years	4.75 (3.94, 5.71)	5.93 (4.64, 7.58)*
3 years	6.02 (4.74, 7.63)	7.52 (5.53, 10.21)*
Main type of sleeping place net was used		
Bed frame (finished)	Ref.	Ref.
Bed frame (sticks)	0.72 (0.60, 0.86)*	0.96 (0.78, 1.17)*
Foam mattress	1.25 (0.92, 1.70)	1.48 (1.06, 2.06) *
Reed mattress	1.69 (1.23, 2.33)*	2.83 (1.98, 4.06)*
Grass mattress	0.57 (0.45, 0.73)*	1.59 (1.20, 2.12)*
Floor with no mattress	3.11 (2.54, 3.82)*	3.68 (2.88, 4.70)*
Others	6.69 (4.07, 10.98)*	5.35 (3.18, 9.01)*
LLIN ever washed		
Yes	Ref.	Ref.
No	0.92 (0.82, 1.04)	1.86 (1.58, 2.20)*
Age household head	1.01 (1.01, 1.02) *	1.00 (0.99, 1.00)
Household head gender		
Male	Ref.	Ref.
Female	1.28 (1.07, 1.53)*	1.10 (0.90, 1.35)
Household head Educational status		
No formal education	Ref.	Ref.
Primary (1–6)	0.93 (0.80, 1.07)	0.89 (0.74, 1.06)
Secondary (7–8)	1.18 (0.94, 1.48)	1.19 (0.90, 1.56)
High school (9–10)	1.20 (0.91, 1.59)	1.19 (0.87, 1.61)
Above high school	1.03 (0.75, 1.42)	1.02 (0.72, 1.45)
Ever exposed to net care and repair messages	0.97 (0.85, 1.11)	
Perception towards net care and repair		
Very positive	Ref.	
Positive	1.05(0.91, 1.22)	1.06 (0.90, 1.25)
Negative	1.33 (1.11, 1.59)*	1.50 (1.21, 1.86)*
Number of rooms used for sleeping	1.21 (1.11, 1.32)*	1.01 (0.90, 1.12)
LLIN to people ratio		
One LLIN for every two people	Ref.	
Less than one LLIN for every two people	0.89 (0.75, 1.05)	1.01 (0.84, 1.22)
More than one LLIN for every two people	1.65 (1.36, 2.00)*	1.31 (1.05, 1.63)*
Wealth quintile		
Lowest	Ref.	Ref.
Second	1.27 (1.05, 1.54)*	1.06 (0.85, 1.33)
Middle	1.29 (1.16, 1.57)*	1.28 (1.02, 1.62)*
Fourth	1.22 (0.99, 1.49)	1.14 (0.88, 1.47)
Highest	2.15 (1.76, 2.63)*	1.86 (1.42, 2.44)*
Study site		
Tigray	9.80 (7.89, 12.12)	5.14 (3.95, 6.68) *
Amhara	3.63 (2.85, 4.62)	4.33 (3.25, 5.77) *
Oromia	3.58 (2.87, 4.46)	1.94 (1.50, 2.50)*
SNNPR	Ref.	Ref.
Malaria transmission setting		
Low (API < 5/1000)	1.42 (1.03, 1.96)*	1.07 (0.72, 1.62)
Moderate (API 5–100/1000)	0.73 (0.53, 0.99)	0.82 (0.58, 1.17)
High (API >= 100/1000	Ref.	Ref.

*p-value < 0.05

Other factors that showed significant associations with LLIN use in the final regression model included: age of LLIN since received by a household, type of sleeping space, washing status of LLIN, perception towards net care and repair, LLIN to people ratio, wealth status, and study site. On the other hand, the factors that were not significantly associated were: brand, age and educational level of household head, exposure to net care and repair messages, and the number of sleeping rooms. Older LLINs had higher odds of not being used compared to newer nets. LLINs that were one (AOR [95% CI] = 3.53 [2.85, 4.37]), two (AOR [95% CI] = 5.93 [4.64, 7.58]) and 3 years old (AOR [95% CI] = 7.52 [5.53, 10.21]) had progressively higher odds of not being used compared to LLINs that were less than 1 year old. LLINs used over sleeping spaces other than mattresses on finished bed frames had a higher chance of not being used. LLINs that were used over floor spaces with no mattress had higher (AOR [95% CI] = 3.68 [2.88, 4.70]) odds of not being used compared to those LLINs used over a finished bed. LLINs that were never washed were highly (AOR [95% CI] = 1.86 [1.58, 2.20]) likely not to be used compared to those that were ever washed. LLINs owned by household heads that had negative perceptions towards bed net care and repair had higher odds (AOR [95% CI] = 1.50 [1.21, 1.86]) of not being used compared to those with positive perceptions. LLINs owned by households that have more than one LLIN for every two people are highly likely (AOR [95% CI] = 1.31 [1.05, 1.63]) not to be used. LLINs owned by households in the middle (AOR [95% CI] = 1.28 [1.02, 1.62]) and highest (AOR [95% CI] = 1.86 [1.42, 2.44]) wealth quintile had higher odds of not being used compared to those in the lowest quintile. The utilization of LLINs significantly varies across study sites. For example, LLINs found in the Tigray study site have higher odds (AOR [95% CI] = 5.14 [3.95, 6.68]) of not being used compared to those in SNNPR. LLINs (Table 2).

## Discussion

This study of the association between levels of LLIN physical damage and reported use revealed that torn LLINs were less likely to be used compared to those not torn. It also identified that LLINs that had been acquired for longer periods, used over sleeping spaces other than finished beds, never washed, in households with negative perceptions towards net care & repair, in households that have more than one LLIN for every two people and owned by households in the middle or highest wealth quintile had lower odds of being used compared to the other categories. Significant variation in the use of LLINs was also identified across study sites.

Torn nets were highly likely not to be used which was in line with other studies done in Ethiopia [7, 8, 12, 23]. This could be due to different reasons. First, household members might perceive that torn nets will provide less or no protection [23]. Second, torn nets might be visually less appalling for use [32]. Regardless of the reason(s), household members are missing the protection that can be gained by using the available LLINs. Studies have reported that significant levels of protection could be gained by using even the torn LLINs due to the insecticidal chemical [33]. Physical decay of LLINs was found to be an important driver of non-use and also attrition [34].

Older LLINs were highly likely not to be used in our context as reported elsewhere [8]. This could be due to the perception that old LLINs are “not good enough” to protect from malaria, which was commonly reported by other studies [21, 23, 35]. The type of sleeping space over which an LLIN is used was an important factor for the utilization of available LLINs. In this study, people sleeping on the floor were likely not to use LLINs. Similar findings were reported in Kenya [36, 37] and Rwanda [38]. One possible reason for this association could be that in rural Ethiopian settings, sleeping on the floor happens in living rooms, that serve other functions during the daytime. Thus, such households might be discouraged from hanging their nets every night before sleeping. LLINs that were never washed were more likely not to be used. Similar findings are also reported by other studies [23]. However, it is difficult to identify the direction of association as LLINs might never be washed because they were not used.

Unlike other studies [16] educational status of the household heads was not found to be a significant predictor of utilization. This could be because the unit of analysis in this study was LLINs and not household members, or other methodological differences. Furthermore, education level might promote LLIN use through better knowledge about malaria, which was not included in this study.

LLINs owned by households that have more than one LLIN for every two people are highly likely not to be used. This could be simply because of having more LLINs to choose from in the house, and those used the night before the survey might be LLINs not included in this cohort study. The variation in utilization of LLINs across study sites could be due to differences in factors not captured by this study including rain season, culture, sleeping arrangement, and availability of other malaria prevention interventions.

This study had several potential limitations worth discussing. (1) The outcome variable was measured based on the utilization status of the LLIN (information obtained verbally from the respondents) the night before the survey and lacked direct observation of LLIN use, which may not reflect accurate utilization. In addition, utilization was measured during the start of the wet season (May and June), hence the identified association might not hold true during other seasons. (2) The study is part of a cohort follow-up study that made four rounds of surveys. This repeated visit of households might affect the regular behaviour of households. (3) The study measured the physical integrity and utilization status of LLINs that were available in households. However, a considerable number of LLINs were lost from households between baseline and subsequent follow-up visits. The physical condition of the removed LLINs might be different from those that remained, and this might have affected the observed association. (4) Some well-known factors for LLIN utilization such as knowledge about malaria and risk perception of getting a malaria infection were not part of the regression models. This is because the parent study did not collect such information. This might affect the strength of the model. (5) This study assessed the association between physical integrity and utilization by making LLINs the unit of analysis, but since the questions were answered by the head of the household, some user-level factors, such as age, gender, and risk perception of specific LLINs users were not included in the model which might have reduced the strength of the model.

In the context of these limitations, the study revealed that LLINs that were torn were more likely not to be used. This, coupled with a high attrition rate and rapid deterioration of the physical integrity of LLINs [39] might reduce protection against malaria, especially in the second and third years following mass distribution campaigns.

## Conclusions and recommendations

LLINs that have some level of physical damage have a relatively higher likelihood of not being used. Community members should be encouraged to keep using their LLINs regularly, even when damaged while promoting the prevention of damage. In addition, further research assessing the effects of sleeping arrangements, risk perceptions, and other factors that might hinder the utilization of available LLINs might be helpful to address the challenge of LLIN utilization.

## Data Availability

All the datasets are available on reasonable request to ACIPH.
